# Gait turning patterns in chronic ischemic stroke males and its relationship to recovery: A cross-sectional study

**DOI:** 10.1097/MD.0000000000017210

**Published:** 2019-09-20

**Authors:** Widjajalaksmi Kusumaningsih, Kevin Triangto, Harris Salim

**Affiliations:** a Department of Physical Medicine and Rehabilitation Cipto Mangunkusumo Hospital; b Neuroscience and Brain Development Cluster, Indonesian Medical Education and Research Institute, Faculty of Medicine, University of Indonesia Jakarta; c Department of Neurology, Faculty of Medicine, Universitas Indonesia, Jakarta, Indonesia.

**Keywords:** chronic ischemic stroke, neuroplasticity, synergy presence, timed up and go, turning

## Abstract

**Introduction:**

Impaired turning patterns have been considered as 1 factor which potentially leads to disability in chronic stroke patients. Mobility comprises 80% of the chief disability, and would eventually lead to falls. Expanded Timed Up and Go (ETUG) is an effective mobility assessment method. It utilizes video recording to analyze the conventional Time Up and Go (TUG) Test components, which includes turning pattern analysis.

**Methods:**

Six healthy males without stroke history and 21 chronic ischemic stroke males (divided into subjects with or without the presence of flexor synergy pattern subgroups) capable of independent ambulation were recruited from Neurology and Medical Rehabilitation Department outpatient clinic. ETUG tests were recorded for each subject and were analyzed thoroughly using a computer program.

**Results:**

Timed Up and Go time was significantly different between the 3 groups (*P* = .001). As compared to control, and synergy absent group, median turning time was highest in chronic stroke patients with presence of flexor synergy by 2786 ms (*P* = .002), but was not significantly different in percentage ETUG (14%, *P* = .939). Further analysis revealed that Brunnstrom stage and number of steps taken for turning significantly affect TUG duration. Other factors such as hemiparetic side, or body height were not significantly associated.

**Discussion:**

The presence of flexor synergy would significantly affect turning time, this would then correlate to the disability of shifting body's center of gravity, as a part of the Stroke core set of International Classification of Functioning, Disability, and Health (ICF).Therefore, stroke patients need to have early ambulatory training regarding pivoting motion rather than solely focusing on straight walking. Instead of hemiparetic side, it is possible that overall turning time is affected by coordination and orientation capability, signifying the importance of cortical plasticity.

## 1 Introduction

Mobility has been the common achievable goal in chronic phase stroke rehabilitation. A Cochrane review had shown that among all the goals that are aimed in their long term rehabilitation, mobility had the most significant improvement.^[1]^ Regaining motor control would also lead to better quality of life in these patients.^[2,3]^ It is true that this goal could be better achieved when accompanied by good training compliance, motivation, number of morbidities, and presence of factors towards neuroplasticity.^[1,4,5]^ Besides quality of life, improving mobility would also significantly reduce their risk of falls, preventing further morbidities, and disabilities.^[6]^

Among the components of mobility, the task of turning during walking generally requires sequential adjustments of vestibular perception, visual function, directional orientation, gait speed alteration, postural control, and a dynamic change in directional forces.^[7–9]^ In stroke patients where spasticity is generally present, these prerequisites of turning would then be impaired, partially or completely, resulting in an abnormal turning pattern.^[6,10]^ It was found previously that stroke patients performing dual tasks would have a great impact on slowing straight walking speed, and increasing stride time, leading towards instability before turning.^[6]^ Coordination control disorder in stroke patients would further detriment the condition, disturbing training compliance, lowering confidence, and finally promoting actual falls or fear of falls.^[9,11]^

Rehabilitation concepts of stroke adopt the utilization of International Classification of Functioning, Disability, and Health (ICF) concept as proposed by the World Health Organization (WHO). The concept then subdivides major health conditions into core sets, incorporating components such as (*b) Body Function*, *(s) Body Structure*, *(d) Activity and Participation*, and *(e) Environmental Factors* that are affected by the discussed condition.^[12]^ ICF research branch had already devised their comprehensive core set for stroke, in which mobility is greatly involved. The basic observation of turning pattern then would relate to several important elements in *(d) Activity and Participation*: *d230 Carrying out daily routine*, *d410 Changing basic body position*, *d450 Walking*, and *d460 Moving around in different locations*.^[9]^ These all corroborate the fact on how crucial is the analysis of turning pattern in chronic stroke patients.

TUG test was initially constructed for geriatric patients, and are commonly used in practice due to its simplicity.^[11]^ A recent systematic review had found that this TUG test is sensitive to be utilized in stroke patients, that it has good reliability and it includes many aspects of basic mobility skills.^[13]^ Further studies on TUG in stroke patients had made it clear that TUG could be used for follow-up studies, as it is exceptional in showing mobility improvements over the weeks of rehabilitation.^[11]^ Expanded Timed Up and Go (ETUG) has also been an added modification to the traditional TUG, and was considered sensitive in recording TUG components in stroke patients.^[10]^ One of its component includes turning pattern analysis, in which instability could be identified as they spend longer time in completing the turn, take 5 or more steps during the turn, and also the absence of pivoting.^[6–8,10]^

The importance of mobility outweighs many other aspects, considering it to be the one to have the most significant improvement in stroke rehabilitation trials.^[1,14]^ Many attempts have been done to predict gait recovery of stroke patients, but among all, time since stroke onset towards rehabilitation initiation had shown weak positive response.^[15,16]^ Topographical identification of stroke lesion though weakly, had also shown relationship to walking parameters. It was shown that non-corticospinal tract lesions (putamen and neighboring structures) could affect walking speed, whereas when corticospinal tract is affected, it will then impact everyday functions of general mobility and gait.^[16]^ There are various measurement scales that are used to assess post stroke motoric scores, however studies had shown that Brunnstrom's motor recovery grading has been widely used to assess the presence of synergy patterns, which is 1 component of motoric recovery.^[15]^ The recovery grading then uses a 7 point hierarchy scale, in which stage 1 appears after stroke onset, that is flaccid stage, stage 2 up to 4 shows the development of spasticity synergy, stage 5 is where voluntary movement starts to dominate the synergy, and finally stage 6 shows how spasticity is no longer present, coordination and voluntary movements are near normal.^[17,18]^ In the original published definition, the stage 7 of Brunnstrom's stage shows the presence of age appropriate normal variety of complex movement patterns, and there is no evidence of functional impairment when compared to the non-paretic limb.^[19]^ This assessment could be done by physician's physical examination, evaluating both spasticity and synergy in both hemiplegic extremities, and its validity has also been published previously.^[15,17]^

Prior studies had demonstrated that several factors are accountable in observing instability during turns, although it was not specified for stroke patients.^[6–8]^ Setting effective turning pattern as a rehabilitation goal would require baseline examination on turning patterns of chronic stroke patients, with analysis on various factors affecting it. With all these kept in mind, this study aims to utilize ETUG to further analyze factors affecting turning pattern in chronic ischemic stroke patients, and simultaneously compare the results with healthy controls. It is hypothesized that stroke patients have multiple factors that will result in significantly longer turning time as compared to healthy controls.

## 2 Materials and methods

### 2.1 Subjects

This cross-sectional study had consecutively recruited chronic male stroke subjects from the Neurology and Physical Medicine and Rehabilitation outpatient clinic in Jakarta, Indonesia, with time ranging from January to August 2018. Sample size was determined with a “rule of thumb” formula for analyzing multivariate regression among the variables, and this then required 20 stroke subjects.^[20]^ These subjects had agreed towards the written informed consent, and consecutive sampling was used for every second patient admitted in the clinic. Recruitment only began after obtaining clearance from Ethics Committee of the Faculty of Medicine, Universitas Indonesia (Protocol Number 18–07–18). Stroke inclusion criteria include: Males with more than or equal to 6 months after the last stroke onset, age of 40 to 65 years old, had MOCA (validated Indonesian form) of 26 or above, and is able to ambulate independently with a minimum motoric score of 3. These patients all had been medically treated with stable medications over 3 months, undergone rehabilitation, and are considered to have stable stroke conditions throughout the course of the research.

Healthy males were also recruited from the community as a control group. The subjects were considered healthy controls as they satisfy the following criteria: have never had stroke episodes, no traumatic brain injury, and are able to ambulate independently. Additionally, they should have no complaints of hip, knee, or ankle pain, and therefore would be safe to classify them as healthy ambulators.

The exclusion criteria for both groups are those subjects with impaired cognition, inability to ambulate independently, and are unwilling to follow the informed consent.

### 2.2 Procedures

All subjects, including healthy controls, will undergo the same test procedures. The first part requires a medical interview with a general practitioner to collect personal data and perform physical examinations. Imaging expertise that had been done previously by radiologist was obtained in order to show which topographical locations were affected by cerebral ischemia during their acute stage. The subjects who require ambulatory devices were also encouraged to equip them during the course of the data collection until further instructions.

**Figure 1:**
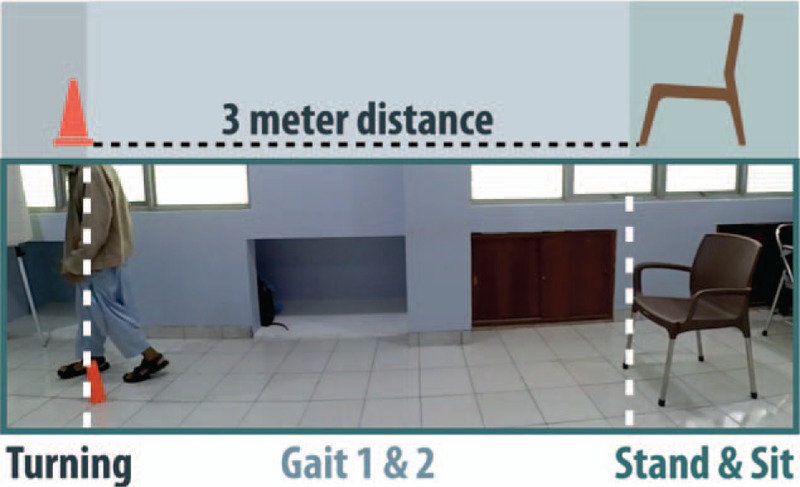
General Setup of Timed Up and Go Test. A standardized chair is placed at the beginning of the setup, and a cone is placed at the end of the 3-meter distance. Three areas are identified as turning area after the cone placement, stand and sit area focusing on the chair, and finally the gait area being the 3-meter distance.

Afterward, the subjects will proceed to a general TUG test, which requires a standardized chair of 46 cm seat height, with arm rest of 67 cm placed at the starting line of the track (Fig. 1). Timer is started with the instruction of “Go”, patient walked a track of 3 meters, turned on a bright colored cone, returned and is stopped as subjects returned to their seat. Instructions and details conform to the original TUG study.^[21]^

The whole process will be recorded using an action camera (YI Action Camera 16 MP) which is placed to view sagittal plane of gait, producing a view shown in Figure 1. Analysis is emphasized on the turning region as the center of gravity (Pelvis) crosses the line (Fig. 1). Three repetitions of TUG trials were recorded and an average value (in seconds) were presented. Turning analysis was presented in milliseconds as they are examined separately on frame-by-frame slow motion speed using a computer program (QuickTime Player 7). The analyses were done by 2 independent reviewers (authors WK and KT) and the final data are composed of the mean values. Both intra- and interrater reliability of ETUG had been published before.^[10]^ Brunnstrom stages were stated in agreement by all the authors, only after the outcomes of TUG and number of steps were done measured (patients were blinded to Brunnstrom subgroups).

### 2.3 Statistical analysis

All numeric data will be analyzed for normal distribution with the help of Saphiro-Wilk Test, and are not considered a normal distribution when *P* is <.05. Data presentation will proceed in the form of mean with its standard deviation if confirmed to have normal distribution, or median with minimum and maximum values when it is not. One-way analysis of variance (ANOVA) test or Kruskal Wallis will be used to compare demographic profile between the 3 cohorts, namely stroke with or without apparent synergy, and healthy controls. Categorical variables such as directional values were presented with percentage, and were analyzed with Fisher Exact Test. The independent t-test was also used to analyze the differences in numeric outcomes for turning parameters. Linear regression was used to perform multivariate analyses. Graphical presentations of the data were also constructed to provide ease in comparison. All statistical tests will be considered significant when *P* is <.05. with 95% confidence interval and power of 80%.^[20]^ These tests will be performed using Statistical Package for the Social Sciences (SPSS) for Macintosh ver. 20.0.

## 3 Results

### 3.1 General characteristics

Twenty-seven males comprising of 6 healthy controls and 21 ischemic stroke patients with or without the presence of flexor synergy pattern and various hemiparesis side were recruited. All numeric data were seen to be normal, except for TUG time (ms), turning time, and the number of steps taken in completing a turn. Power analysis was done on the stroke subjects in this study, and the difference yielded power (1-β) of 0.96. The mean age of both groups was seen to be similar, 49.00 ± 6.42 year for control and stroke 55.67 ± 7.54 years, with no statistically significant difference between them (Table 1). Anthropometric values such as weight, height, and Body Mass Index (BMI) were also not different significantly. In general, all the subjects were in pre-obesity phase. The median TUG values were significantly different, stroke patients taking longer time (12,078 ms) as compared to healthy controls (9884 ms). Cortical ischemic seem to predominate the stroke subjects (71.43%) over the subcortical lesion (28.57%), and none of the samples suffer from brainstem lesion.

**Table 1:**
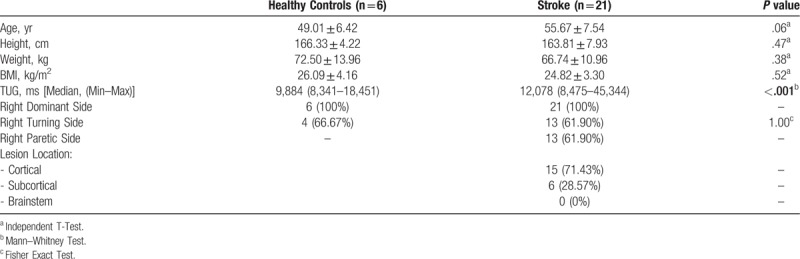
General subject characteristics.

**Figure 2:**
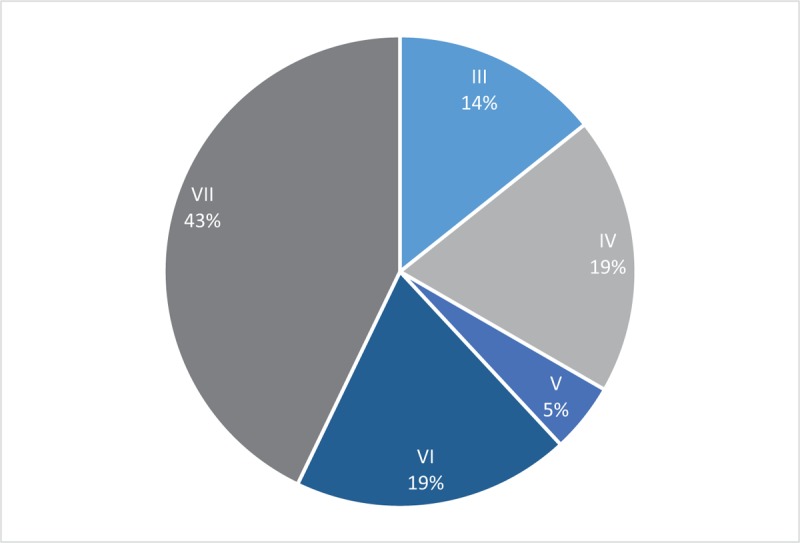
Brunnstrom Staging of the Stroke Subjects (n = 10). Brunnstrom stages are shown on each chart ranging from 3 to 7, each with proportions for the whole stroke study population.

Brunnstrom staging was graded and is displayed on a pie chart (Fig. 2), depicting that among the 21 chronic stroke patients, 28% still had apparent synergy pattern (Brunnstrom stage 3–5), and majority had regained motor control (Brunnstrom stage 6–7).

### 3.2 Turning pattern profile

**Figure 3:**
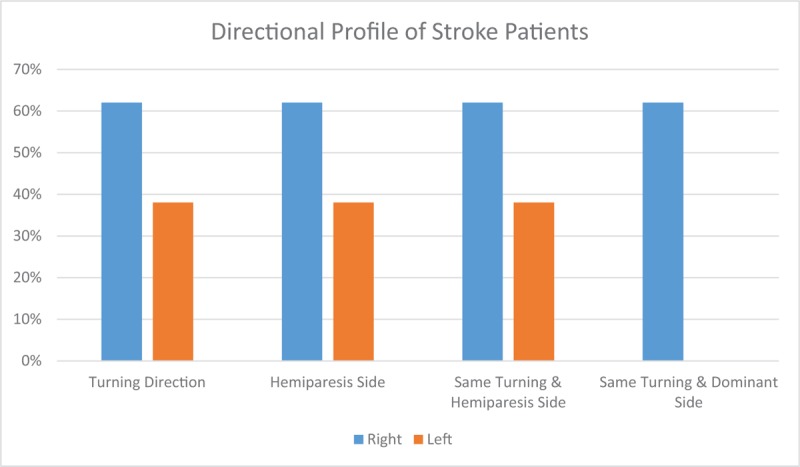
Directional Profile of the Stroke Subjects (n = 10). Turning direction and hemiparetic side are displayed in the graph. Same turning and hemiparetic side, with same turning and dominant side are displayed with only right side profiles.

All subjects were also found to be of right dominant side. As for turning direction, 8 patients in the stroke group chose left turning direction, while all others had right turning direction (Table 1). Roughly 62% of the stroke patients were of right hemiparesis. Turning direction was not found to be associated with hemiparetic side, or dominant side (Fisher exact test are all >.05, Fig. 3).

### 3.3 Comparison between the groups

A comparison in the presence of synergy subgroups in stroke patients was performed, and several turning parameters were compared together with control. Weight and height were similar between the groups (Table 2).

**Table 2:**
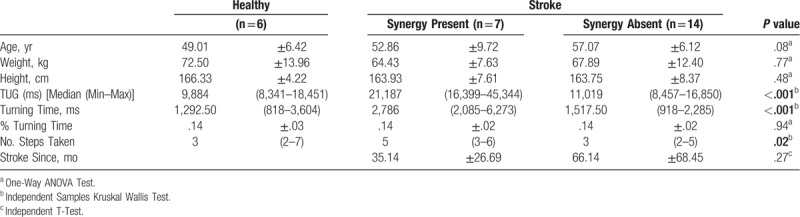
Turning parameters between groups.

Median TUG time was seen to be significantly different, in which presence of synergy was the longest 21,187 ms, followed by absence of synergy 11,019 ms, and the fastest was healthy controls 9884 ms. Turning time, measured in milliseconds also had the same sequence, the longest being presence of synergy was 2786 ms. The number of steps similarly followed the sequence, with the presence of synergy taking 5.00 steps, being significantly different with the other 2 groups. These turning parameter comparisons were all significantly different between the groups (Table 2). Interestingly, when turning time was converted into percentage, the pattern did not persist. That it was seen how the 3 groups all approximately requires 14% of their whole TUG to perform a successful turn.

The duration of stroke since the first onset was expressed in months. Synergy absent group has observable difference as they had longer months since onset (66.14 ± 68.45 months), while it was half the duration in presence of spasticity (35.14 ± 26.69 months). However, the duration difference was proven to not be significantly different (Table 2).

**Figure 4:**
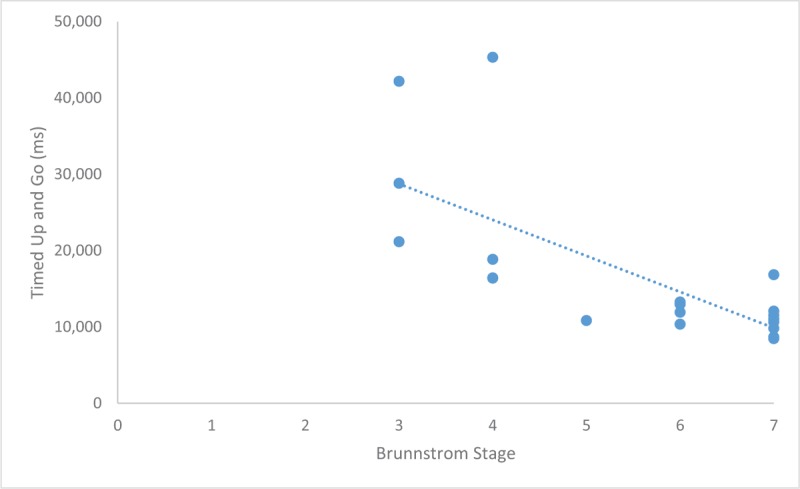
Correlation of Brunnstrom Staging and Mean Timed Up and Go duration. The scatterplot shows a constant significant inverse correlation (r = −.508, *P* = .019) between mean timed up and go with Brunnstrom staging.

**Figure 5:**
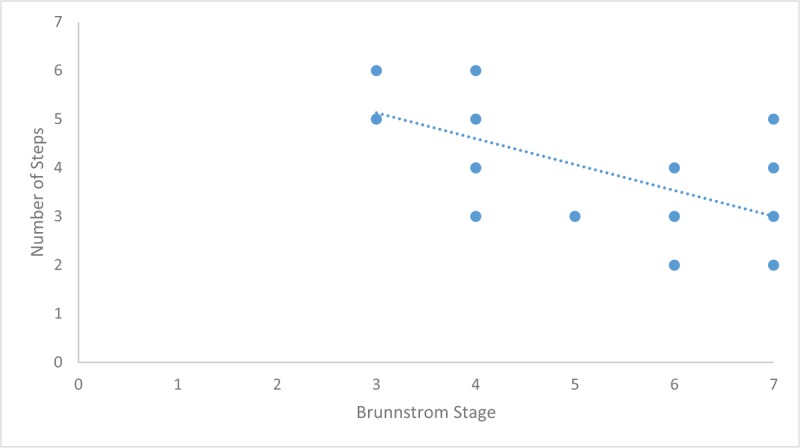
Correlation of Brunnstrom Staging and Number of Steps Taken during a Turn. The scatterplot shows a constant significant inverse correlation (r = −.746, *P* = .001) between number of steps and Brunnstrom staging.

Further analysis was done on the Brunnstrom stage (Figs. 4 and 5). Spearman correlations were found to be significant for both TUG (r = −.508, *P* = .019) and number of steps taken for completing a turn (r = −.746, *P* = <.001), both demonstrated a strong inverse relationship to Brunnstrom. These values and directional relationship were then carried out for univariate linear regression analysis (Table 3). Although both showed significant relationship, it was clearly shown that TUG had a better variance (R^2^ = 52%, Fig. 4) as compared to steps taken for turn (R^2^ = 36%, Fig. 5). Beta coefficient for Mean TUG was increased with Brunnstrom stage by −4718.96 (95% CI −6,882.76 to −2,555.17), and similarly decreased number of steps with inclination in Brunnstrom stage by −0.53 (95% CI −.85 to −.21).

**Table 3:**
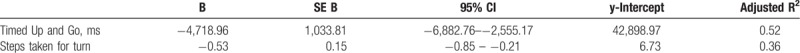
Univariate linear regression analysis for brunnstrom staging to TUG profile and turning profile in ischemic stroke patients.

## 4 Discussion

This study had proven how ETUG were sensitive to changes in turning profile of stroke patients when compared to healthy controls. By using video observation, the reviewers could indicate direction, number of steps, and duration of turning. The investigation was further continued and compared to the side of hemiparesis, dominant side, and percentage time taken for overall ETUG. Stroke patients significantly were observed to have longer duration, more steps, and different percentage proportion. Acknowledging those differences in stroke patients, the study had also found interesting results on the number of steps taken to complete a turn. The number of steps was 1 of the reasonable differences in both mean TUG and stages of recovery (Brunnstrom) in stroke patients, in which lower number of steps signify better turning profile for these subjects.

There are multitude of impairments that precede the disability in mobility. Some of them include visuospatial neglect, hypoesthesia, hemiparesis spasticity, and even cognitive decline.^[22]^ Many studies had shown that spasticity was 1 of the main contributors towards longer turning time.^[22–24]^ The presence of spasticity would bring forth the classical hemiparetic gait of stroke patients, leading to a circumduction motion of the lower limb as it pertains the extensor synergy. This significantly increases mediolateral locomotor trajectories of the center of mass along the TUG track, especially during forward gait and turning.^[24]^ Turning itself requires a sequence of tasks such as visuospatial observation on turning indicator, deceleration of forward gait, execution of pivot motion on 1 side, and also beginning acceleration towards the next forward gait as the turn is completed.^[25]^ Incapability of stroke patients to perform all these then leads to instability.^[24,25]^ Among all these tasks, execution of pivot motion would require the highest control on neuromuscular coordination, as it first requires a different group of muscles as compared to a normal forward gait.

Pivoting requires the action of an ipsilateral gluteus medius muscle as a controller on the single leg support (i.e., the leg closest to the turning point), providing mediolateral stability. Whereas hip extensors are required for effective weight transfer to contralateral leg.^[26]^ Weakness of gluteus medius muscle will be seen as positive Trendelenburg sign, and will cause instability during the turn. The spasticity of gluteus maximus as a powerful hip extensor will result in the emergence of hemiparetic gait, subsequently shrouding the weakness of gluteus medius. Previous studies had mentioned that turning direction on hemiparetic stroke patients had no effect on the total TUG performance.^[10,22,23,25]^ Another study had seen how turning in stroke patients towards their hemiparetic side would result in shorter stride length, longer stride time, or pauses more frequently as compared to the healthy controls.^[27]^ Turning strategies as had been described by the earlier study, was not always discussed in later studies, and thus precipitated in a wide range of turning parameters across studies.^[8]^ Additionally, the presence of synergy (Brunnstrom stage 3–4) had been shown to correlate with lateral pelvic tilt during stationary position,^[28]^ it could be then inferred that Brunnstrom stage would affect turning steps, which is adapted as turning strategies by the patients, presented in Figure 5. Although stride length could not be measured with the video equipment used in this study, it could be translated to a significant increase in the number of steps for stroke patients, especially when spasticity is present, and this result is similar as the previous studies.^[22,27,29]^

The percentage comparison in turning was not seen to be significantly different. This would show how turning may be a constant factor in the whole TUG performance which could be predictive in stroke patients to oversee recovery. The reference study had shown larger percentage turning time comparison between healthy and stroke being 16.14 ± 2.58% and 15.45 ± 3.18% without reaching statistically significant difference; whereas the present study had shown 14% to be a common number in between groups. Presence of synergy as graded by Brunnstrom staging also shows obvious differences in the TUG performance. It is still speculated whether onset after stroke is directly related towards Brunnstrom staging, however, it could be seen that an individual would adapt a walking pattern, and with time it eventually translate towards inclining gait speed.^[1,15,17]^ There are still very few studies on stroke patients that utilized ETUG, but across the earlier studies, it was seen that stroke patients generally spend lower percentage turning time as compared to normal. It was previously published that although chronic stroke may differ in lesion location, their long term recovery is not directly related to where the location is.^[5,16]^ It would rather be the presence of other factors such genetic variation, which then translates to the concentration of Brain Derived Neurotrophic Factor (BDNF), a neurotrophin in the systemic circulation, and its possibility to be correlated to neuroplasticity potential of each individual.^[3,30]^ Nonetheless it should be kept in mind that chronic stroke patients recruited in this study had an onset of 6 months or above, therefore it is likely that they had already achieved an advanced walking pattern which most probably would be similar to their normal pace, be it with the presence of synergy or not.

It is also observed previously that hemiparesis side would not affect overall TUG performance. This protocol used in this study observes the preferred turning side instead of providing instructions of where to turn in order to see which visuospatial direction is more dominant. Our results revealed that almost all subjects performed right turn, and this was not associated with either hemiparetic side or dominant side. Interestingly, 1 recent study had shown how turning towards unaffected side would result in significantly longer turning time, owing to the fact that the hemiparetic limb needs to cover longer distances.^[31]^ Hence it is still unknown on what factors that could predict turning direction, such as unilateral limb strength, daily gait exercise technique or post-stroke visuospatial orientation, turning strategies, all of which require further observations.^[8,24]^ In addition to those factors, our study was also limited in displaying gender biomechanical differences in gait. Despite previous studies’ report on no significant differences between gender for TUG profile, it could be observed that there are several aspects that may affect biomechanical gait differences between them.^[10,25,26]^ Some of those aspects include broader hip that affect center of mass sway, knee alignment, and muscle strength which relates to footwear preferences. Further studies could be extended to analyze them in order to allow better generalizations of the results in stroke patients.

In spite of the small sample, this study had successfully shown how chronic stroke had recognizable difference in turning pattern, which was detected by ETUG observations. The presence of synergy was also seen as a prominent aspect that could reduce TUG performance, especially in slowing turning duration by increasing number of steps to complete the turn. Identifying these problems would assist medical rehabilitation specialist in setting tailor-made programs to exercise individual muscles which would differ depending on many factors, including frequently preferred turning direction, presence of synergy and hemiparetic side.

## 5 Conclusion

These results then confirmed how turning pattern in chronic stroke is different from their age, gender, and height-matched healthy controls. Significantly longer turning duration is expected, which could be caused by the presence of synergy, and overall yielded an increased number of steps to complete a turn. All these then presents a powerful barrier experienced by stroke patients, exposing them with higher risk of falls. Lesser number of steps taken for turns then demonstrates a better turning profile and subsequently suggests promising observations towards neuroplasticity progresses in stroke patients.

## Author contributions

**Conceptualization:** Kevin Triangto, Widjajalaksmi Kusumaningsih, Harris Salim.

**Data curation:** Widjajalaksmi Kusumaningsih, Kevin Triangto.

**Formal analysis:** Widjajalaksmi Kusumaningsih, Kevin Triangto, Harris Salim.

**Funding acquisition:** Widjajalaksmi Kusumaningsih.

**Investigation:** Widjajalaksmi Kusumaningsih, Kevin Triangto, Harris Salim.

**Methodology:** Widjajalaksmi Kusumaningsih, Kevin Triangto, Harris Salim.

**Project administration:** Widjajalaksmi Kusumaningsih, Kevin Triangto, Harris Salim.

**Resources:** Kevin Triangto, Harris Salim.

**Software:** Kevin Triangto.

**Supervision:** Widjajalaksmi Kusumaningsih, Harris Salim.

**Validation:** Widjajalaksmi Kusumaningsih, Kevin Triangto.

**Visualization:** Widjajalaksmi Kusumaningsih, Kevin Triangto.

**Writing – original draft:** Kevin Triangto.

**Writing – review & editing:** Widjajalaksmi Kusumaningsih, Kevin Triangto, Harris Salim.

Kevin Triangto orcid: 0000-0003-3239-3542.

Widjajalaksmi Kusumaningsih orcid: 0000-0002-3203-8455.

Harris Salim orcid: 0000-0002-4271-2933
